# Lysosomes, curcumin, and anti-tumor effects: how are they linked?

**DOI:** 10.3389/fphar.2023.1220983

**Published:** 2023-07-07

**Authors:** Qian Shen, Xue Pan, Yi Li, Junchen Li, Chuanlong Zhang, Xiaochen Jiang, Fudong Liu, Bo Pang

**Affiliations:** ^1^ Guang’anmen Hospital, China Academy of Chinese Medical Sciences, Beijing, China; ^2^ Tianjin University of Traditional Chinese Medicine, Tianjin, China

**Keywords:** curcumin, lysosome, tumor, proliferation, metastasis, drug resistance

## Abstract

Curcumin is a natural active ingredient from traditional Chinese medicine (TCM) that has multi-target characteristics to exert extensive pharmacological activities and thus has been applied in the treatment of various diseases such as cancer, cardiovascular diseases, nervous system, and autoimmune disorders. As an important class of membranous organelles in the intracellular membrane system, lysosomes are involved in biological processes such as programmed cell death, cell metabolism, and immune regulation, thus affecting tumor initiation and progression. It has been shown that curcumin can modulate lysosomal function through the aforementioned pathways, thereby affecting tumor proliferation, invasion, metastasis, drug resistance, and immune function. This review briefly elaborated the regulatory mechanisms of lysosome biogenesis and summarized curcumin-related studies with its anti-tumor effect, providing a reference for the clinical application of curcumin and anti-tumor research targeting lysosomes.

## 1 Introduction

Curcumin, an orange-yellow polyphenolic compound originating from TCM, has attracted much attention owing to its anti-inflammatory, anti-bacterial, anti-oxidant, and other biological activities. Many studies have focused on the anti-tumor activity of curcumin. It has been illustrated that curcumin can exert an anti-tumor effect by modulating growth factors, enzymes, transcription factors, kinases, inflammatory cytokines, and pro- and anti-apoptotic proteins, yet in-depth research is still in infancy (Giordano and Tommonaro, 2019). Lysosomes are a kind of organelle that can eliminate unnecessary biomacromolecules, senescent organelles, and senescent, damaged, and dead cells, provide a defense, and offer nutrients to the cells. With the in-depth investigation, lysosomes have been a research hotspot in the fields of pharmacy, chemistry, and life sciences. Numerous studies have shown that the functional state and spatial distribution of lysosomes are closely related to the initiation and progression of tumors, and targeting lysosomes provides a novel insight into the diagnosis and treatment of tumors. This paper systematically summarized the anti-tumor mechanisms of curcumin targeting lysosomes to provide important guidelines for clinical diagnosis and treatment of tumors and in-depth elucidation of the pharmacological effects of curcumin.

## 2 Medicinal properties of curcumin

**FIGURE 1:**
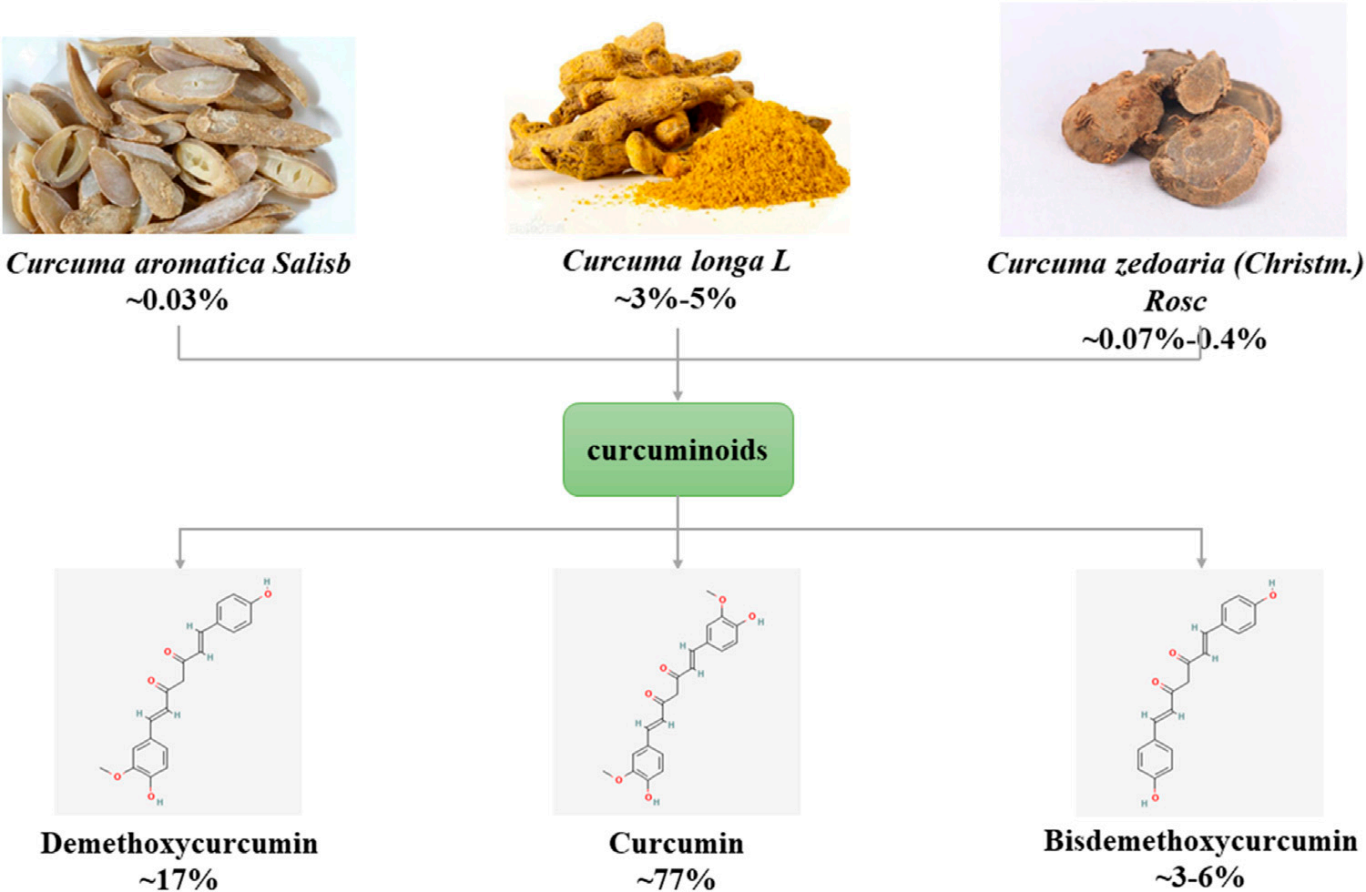
Source and classification of curcuminoids.

The main source of curcumin (1,6-Heptadiene-3,5-dione,1,7-bis(4-hydroxy-3-methoxyphenyl)-) is the tuberous root or rhizome of plants of Zingiberaceae and Araceae (such as Curcumae Radix, Curcumae Longae Rhizoma, Curcumae Rhizoma), first isolated by Vogel and Pelletier in 1815. Curcumin is a rare pigment with diketone structure in the Plantae. It is orange yellow crystalline powder, slightly bitter taste, insoluble in water, chemical formula C21H20O6. Curcumin is a type of curcuminoids, and its natural homologues include demethoxycurcumin and bisdemethoxycurcumin (Gopi et al., 2017) (Figure 1). Curcumin has anti-inflammatory, anti-diabetic, anti-tumor and anti-aging therapeutic potential and is widely used in various diseases such as cancer, cardiovascular disease, neurological and autoimmune (Raghav et al., 2021).

### 2.1 Pharmacokinetics of curcumin

Curcumin’s poor absorption *in vivo*, rapid metabolism, low bioavailability, and short half-life in the gastrointestinal tract limit its efficacy (Anand et al., 2007). The main modes of curcumin administration include oral administration and injectable administration, and the bioavailability of the two differs considerably. The study showed that the serum level of curcumin was 0.36 ± 0.05 μg/mL in rats after intravenous injection of curcumin 10 mg/kg, while the serum level of the drug was only 0.06 ± 0.01 μg/mL after oral administration of 50 times the dose of curcumin (Yang et al., 2007). Zhang et al. studied the pharmacokinetics and absolute bioavailability of curcumin in rats after intragastrical administration, intraperitoneal administration and sublingual. The experimental results showed that the metabolic process of curcumin in rats in the three administration routes was consistent with the two-compartment model, with t1/2 of 159.28, 90.79 and 11.96 min, respectively. The area under the drug-time curve (AUC0-∞) was 86.36, 73.39 and 104.62 mg min·L^−1^, respectively. The absolute bioavailability of intraperitoneal administration was 35. 07%, while that of intragastrical administration was only 4.13% (Zhang et al., 2011). The low oral bioavailability of curcumin may be due to a first-pass effect and a certain degree of intestinal metabolism, especially the high binding rate from glucuronidation and sulfation into its water-soluble metabolites and excretion in the urine, so that the drug concentration in the plasma is low (Sharma et al., 2007). Most of the research on curcumin in recent years has been looking to overcome the problem of low bioavailability of curcumin. Researchers have established nanoparticles, liposomes, micelle, phospholipid complexes and other methods to prolong the residence time of curcumin *in vivo*, increase the biological permeability of curcumin, slow down the metabolic process of curcumin, and finally improve the bioavailability of curcumin (Abd El-Hack et al., 2021). For example, curcumin loaded nanofibers can improve sustained release behavior and bioavailability and inhibite MCF-7, HepG2 and L929 cell lines. The active targeting of liposomes can improve the solubility, stability and drug loading efficiency of curcumin and the toxicity to tumor cells (Zhang et al., 2019).

After entering the body, curcumin can be distributed in plasma, liver, kidney and brain tissue, indicating that curcumin can cross the blood-brain barrier (Shehzad et al., 2010). The metabolic sites of curcumin are mainly in the intestine and liver. After oral administration, curcumin is first absorbed and metabolized from the intestine, then enters the systemic blood circulation, and finally is taken to the liver for extensive metabolism, and curcumin undergoes a complex transformation during absorption through the intestine (Wang and Qiu, 2013). The metabolism of curcumin *in vivo* may include two stages: reduction, dehydroxylation reaction and glucuronidation, sulfation. The metabolites of curcumin are related to the mode of administration, and when administered orally, curcumin is metabolized primarily to glucuronates and glucuronate/sulfate conjugates (Asai and Miyazawa, 2000). However, when administered intravenously or intraperitoneally, curcumin is metabolized to tetrahydrocurcumin, hexahydrocurcumin, and octahydrocurcumin (Ravindranath and Chandrasekhara, 1981). Some researchers also believe that curcumin-glucuronic acid, dihydrocurcumin-glucuronic acid, tetrahydrocurcumin-glucuronic acid and tetrahydrocurcumin-glucuronic acid are the main metabolites of curcumin *in vivo* (Pan et al., 1999).

There are no studies on the excretion kinetics of curcumin, and similar to most drugs, curcumin and its metabolites are mainly excreted through the kidneys and bile, while quercetin can modulate the pharmacokinetics of curcumin *in vivo* and promote the excretion of curcumin via bile acids (Kim et al., 2012).

### 2.2 Safety evaluation of curcumin

Previous studies have reported that curcumin has no obvious acute toxicity, subchronic toxic damage, and no potential mutagenic or teratogenic effects in animal toxicity experiments, and few adverse reactions have been reported, indicating that curcumin has high safety (Zhou et al., 2019). Nelson et al. found that a single oral high dose of curcumin 12 g/d still did not produce any side effects (Nelson et al., 2017). According to the reports of the Joint FAO/WHO Expert Committee on Food Additives and European Food Safety Authority the Acceptable Daily Intake values for curcumin are 0–3 mg/kg (Kocaadam and Şanlier, 2017). In a phase I clinical trial, Sharma et al. gave oral curcumin capsules (0.45–3.6 g/d) to patients with advanced colon cancer. Two patients had diarrhea after 1 and 4 months of treatment, respectively, and another patient had nausea, and the symptoms spontaneously relieved after continued treatment. In addition, elevated serum alkaline phosphatase levels were found in 4 patients and elevated serum lactate dehydrogenase levels in 3 patients on blood tests (Sharma et al., 2004). In a dose-escalation study of curcumin conducted by Lao et al., two patients developed diarrhea at 1,000 mg and 12,000 mg, one patient developed headache at 4,000 mg, two patients developed yellow stools and rash at 8,000 mg, respectively, and two patients developed yellow stools and headache at 10,000 mg, respectively, all with toxicity grade 1 (National Cancer Institute, Common Toxicity Criteria v.2.0) (Lao et al., 2006).

## 3 Regulatory mechanisms of lysosome biogenesis

In 1949, de Duve first demonstrated the presence of lysosomes as an organelle in the study of the distribution of sugar metabolic enzymes in rat liver tissues, and collaboratively, de Duve and Novikoff first observed specific morphological structures of lysosomes under an electron microscope in 1955. Lysosomes are monolayer-enveloped vesicular organelles containing more than 60 hydrolytic enzymes. Lysosomes are heterogeneous with different morphology and sizes and contain different enzymes in different cells or the same cell. Lysosomes can be assigned into primary lysosomes, secondary lysosomes, and residual bodies according to their different functional stages. Secondary lysosomes can further be divided into autolysosomes and heterolysosomes, depending on the complex formed after the fusion of primary lysosomes with autophagic or heterophagic vacuoles. After digestion by secondary lysosomes, a portion of small-molecule substances can still be metabolically utilized by cells, while the undigested substances are excreted out of the cell in the form of residual bodies.

**FIGURE 2:**
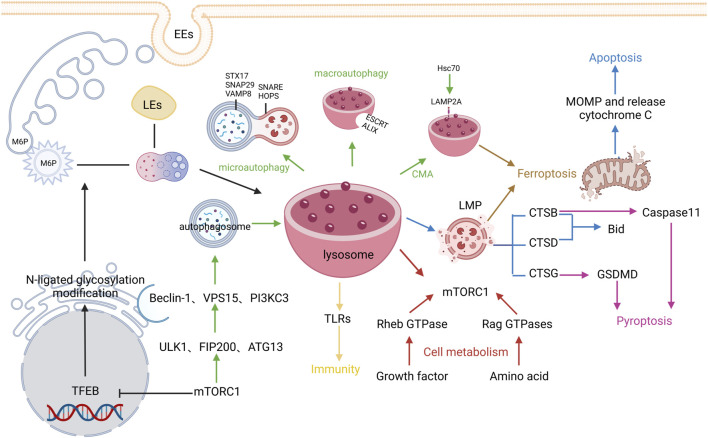
Regulatory mechanisms of lysosome biogenesis. The black arrows represent Lysosome biogenesis, the green arrows represent Autophagy, the blue arrows represent Apoptosis, the pink arrows represent Pyroptosis, the brown arrows represent Ferroptosis, and the red arrows represent cell metabolism, The yellows arrow indicate immunity.

The optimal pH condition for diverse lysosomal hydrolases is around 3.5–5.5, so the lysosomal interiors must be consistently maintained in the acidic environment. The acidic environment within lysosomes is mainly maintained by vacuolar H^+^-ATPase (V-ATPase), chloride channels, and ion transporters in the lysosomal membrane. V-ATPase is an ATP-driven proton pump composed of the cytosolic V_1_ domain and the integral V_0_ domain that can not only maintain the internal acidic environment of lysosomes but also participate in the regulation of cellular metabolism (Cotter et al., 2015; Holland et al., 2020). Different from the internal acidic environment of lysosomes, the pH value in the cytoplasm is generally around 7.0, and the lysosomal membrane plays a crucial role in maintaining this concentration difference of H^+^ inside and outside the lysosomes. The lysosomal membrane contains a single phospholipid bilayer and integral and peripheral proteins. Lysosomal membrane proteins mainly consist of trafficking and fusion machinery proteins such as soluble NSF attachment protein receptors (SNAREs) and targeting GTPases (RABs), structural proteins such as lysosome-associated membrane protein-1 and -2 (LAMP-1 and LAMP-2), and transporters such as NPC Intracellular Cholesterol Transporter 1 and lysosomal amino acid transporter 1. Among them, LAMP-1 and LAMP-2 are highly glycosylated transmembrane proteins that account for approximately 50% of total lysosomal membrane proteins (Eskelinen, 2006). The formed “glycocalyx” has been considered to protect the membrane from degradation by lysosomal enzymes (Settembre et al., 2013). Additionally, lysosomes contain rare negatively charged lipid bis(monoacylglycerol)phosphates, which can serve as one of their hallmarks (Rudnik and Damme, 2021). It is traditionally believed that lysosomes, the “degradation station” for cellular cargoes such as proteins and fat, can degrade metabolic wastes in the cells *via* endocytic, phagocytic, and autophagic pathways (Saftig and Klumperman, 2009). In recent years, with the development of cell biology and physiology, the functions of lysosomes have been more and more expanded, which are regarded to be closely linked to programmed cell death, cell metabolism, cell immunity, etc (Zhang et al., 2021) (Figure 2)

### 3.1 Lysosome biogenesis

The lysosome biogenesis involves two pathways: biosynthetic pathway and endocytic pathway. The biosynthetic pathway is mainly regulated by the microphthalmia/transcription factor E (MiT/TFE) protein family, in which transcription factor EB (TFEB) is being studied mostly. Generally, TFEB can be dephosphorylated and activated by the phosphatases calcineurin (Medina et al., 2015) and protein phosphatase 2 (Medina et al., 2011) or phosphorylated and thus inhibited by the atypical serine/threonine kinase mechanistic target of rapamycin complex 1 (mTORC1) (Martina et al., 2012) and serine/threonine kinases such as mitogen-activated protein kinase 1 (Settembre et al., 2011), protein kinase B (Palmieri et al., 2017), glycogen synthase kinase 3β (Li et al., 2016), and mitogen-activated protein kinase 3 (Hsu et al., 2018). The activated TFEB can transcribe the coordinate lysosomal expression and regulation gene, which then encodes proteins related to lysosomal structure and function, mainly including multiple soluble lysosomal hydrolases (also referred to as acid hydrolases) and integral lysosomal membrane proteins (LMPs). These proteins are first synthesized in the rough endoplasmic reticulum (ER) and then modified by N-linked glycosylation. Next, they are transferred to the Golgi, modified by N-acetylglucosamine phosphotransferase and phosphoglucosidase, and captured by sorting receptors. The common sorting receptors mainly include mannose-6-phosphate receptors, sortilin, and lysosomal integral membrane protein 2, and different sorting receptors are involved in the transportation of different types of soluble lysosomal hydrolases and LMPs (Hunziker and Geuze, 1996). Lastly, clathrin/AP-coated vesicles are formed after budding on the Golgi apparatus and transported out. Meanwhile, early endosomes are derived from cell membranes *via* endocytosis and their interiors can be gradually acidified through V-ATPase, simultaneously fused with the same type of endosomes to increase their volume, and gradually transformed into late endosomes (LEs). The internal environment of LEs is further acidified to release RAB5 and capture RAB7 (Rink et al., 2005; Saftig and Klumperman, 2009). Subsequently, clathrin/AP-coated vesicles are mutually integrated with LEs, receptors are recycled, and finally mature primary lysosomes are formed after the dephosphorylation of lysosomal enzymes. Given these findings, lysosome formation is a finely-tuned dynamic biological process.

### 3.2 Mechanisms of lysosomes in regulating programmed cell death

During the investigation of programmed cell death, numerous investigators have gradually identified the roles of lysosomes in cell death. As a digestive center, lysosomes can participate in the regulation of cell autophagy, apoptosis, pyroptosis, and ferroptosis through many pathways.

#### 3.2.1 Autophagy

Autophagy is not active but only activated in response to stress stimulation (including nutrient deprivation, environmental toxicants, infection, and oxidative stress) (Kroemer et al., 2010; D'Arcy, 2019). Autophagy can be mainly classified into three types: macroautophagy, microautophagy, and chaperone-mediated autophagy (CMA) (Nie et al., 2021).

During macroautophagy, autophagosomes are formed with the membrane-enclosed degradable substances derived from the ER, Golgi apparatus, or cytoplasmic membrane, followed by fusion with the lysosomes and degradation of their contents. The aforementioned TFEB is a master transcriptional regulator of lysosomal autophagy in addition to its important role in lysosomal biogenesis (Chen M. et al., 2021). Under the stress condition, the mTORC1 expression is inhibited, and TFEB is dephosphorylated, thereby promoting its nuclear translocation and inducing cell autophagy. Autophagy requires the formation of autophagosomes, which primarily occurs at the ER *via* an omegasome (Medina, 2021). In addition, reduced mTORC1 expression stimulates the autophosphorylation of unc-51 like autophagy activating kinase-1 (ULK1) and the formation of a complex with focal adhesion kinase family interacting protein of 200 kDa and autophagy-related 13 (ATG13), thus accelerating the formation and elongation of omegasomes (Jung et al., 2009). Meanwhile, ULK1 also phosphorylates Beclin-1 to activate the Class III phosphatidyl inositol 3-kinase (PI3K) activity complex containing Beclin-1, vacuolar protein sorting15, and PI3KC3 and promotes the elongation of autophagosomal membranes by recruiting ATG WD repeat domain phosphoinositide-interacting proteins (Kihara et al., 2001). After endosomal sorting complex required for transport (ESCRT)-III-mediated phagophore closure, mature autophagosomes are formed (Zhen et al., 2020). Multiple mechanisms can promote the fusion of autophagosomes with lysosomes; for instance, insertion of SNARE into the membrane induces the formation of alkaline phosphatase tethering complex (Fader et al., 2009); tectonin beta-propeller repeat containing 1 interacts with phosphatidyl inositol triphosphoric acid and ATG12-ATG5 conjugates (Chen D. et al., 2012); RAB7 (Jäger et al., 2004) and syntaxin 17 (STX17)-synaptosome associated protein 29- vesicle associated membrane protein 8 complex (Liu et al., 2015). After the autophagosomes are fused with the lysosomes, the substances will be scavenged and recycled by lysosomal enzymes.

Microautophagy is mainly realized through three pathways, invaginations of lysosomal membranes, protrusion of lysosomal membranes, and invaginations of endosomes; the lysosomal membrane invagination occurs in the surface of the lysosomal membrane by which cytosolic proteins are sequestrated with other cytosolic components through the coordination of interacting protein X and ESCRT (vacuolar protein sorting 4 Homolog A and B, and tumor susceptibility 101) (Tekirdag and Cuervo, 2018; Mahapatra et al., 2021). CMA mostly occurs in proteins containing the KFERQ sequence (Kaushik et al., 2011). These proteins are transferred to the lysosomal interiors *via* the lysosomal transporter LAMP2A by binding to the molecular chaperone heat shock cognate 71 kDa protein (Hsc70) (also referred to as HSPA8 in humans) and thus degraded (Kaushik and Cuervo, 2018).

#### 3.2.2 Apoptosis

Apoptosis is an autonomous and orderly cell death controlled by genes. When an apoptotic cell is recognized, the membrane-bound phagosome enveloping the target cell is formed after phagocyte pseudopod extension and fusion. Separation of phagosome from the plasma membrane by phagosome scission initiates the maturation process, which is controlled by the serial actions of RAB GTPases, resulting in the formation of phagosomes; subsequently, phagocytes will rapidly clear the apoptotic cells *via* lysosome-mediated degradation, ultimately preventing inflammation and controlling immune responses (Yang and Wang, 2017).

Many classical apoptotic stimuli, including tumor necrosis factor receptor family, p53 activation, interleukin 1, and growth factor deprivation, can trigger increased lysosomal membrane permeability. In addition to serving as a mere executor of death in the regulation of apoptosis, lysosomes can also function as initiators and executors to mediate apoptosis. Cells have distinct morphological consequences upon lysosomal membrane permeabilization, and the release of various lysosomal enzymes into the cytoplasm triggers intrinsic apoptosis and apoptosis-like responses (Repnik et al., 2014). Overall destruction of lysosomes causes rapid necrosis in cells. Cathepsin (CTS) released after lysosomal membrane permeabilization can participate in the execution of the classical apoptotic program. The released CTSB and CTSD cleave the pro-apoptotic protein Bid and cause the translocation of its hydrolysates, and the activated Bid induces the secretion of mitochondrial outer membrane permeabilization and cytochrome c, initiating the intrinsic apoptotic pathway (Boya and Kroemer, 2008), which is known as “lysosome-mitochondrial pathway” to mediate apoptosis. In response to excessive lysosomal enzymes, receptor-interacting serine/threonine protein kinase 1 and 3 can induce necroptosis (Vandenabeele et al., 2010). Furthermore, the lethal effect of LMP and CTS in the cytoplasm not only activates the mitochondria-dependent intrinsic apoptotic pathway but also mediates apoptosis in a mitochondria-independent pathway; for instance, CTS in lysosomes play a pivotal role in inflammatory response-related apoptosis.

#### 3.2.3 Pyroptosis

Pyroptosis is a programmed cell death manifested with constant cell swelling until the rupture of cell membranes, leading to the release of cellular contents, which in turn activates a strong inflammatory response. Pyroptosis mainly requires inflammasome-mediated activation of caspase-1, which, on the one hand, leads to gasdermin D (GSDMD) cleavage and dissociation of GSDMD N-terminus from the GSDMD C-terminus, causing cell perforation, and on the other hand, stimulates the extracellular release of interleukin (IL)-1β and IL-18, triggering cascade amplification of inflammatory response (Kovacs and Miao, 2017; Burdette et al., 2021). CTSG can efficiently cleave GSDMD to generate the GSDMD-N fragment, inducing pyroptosis (Burgener et al., 2019). Caspase-1 activation is mainly dependent on the activation of nucleotide binding oligomerization domain-like receptors, which not directly binds to and activates caspase-1 but recruits and induces caspase-1 activation by constructing a multi-molecule platform called the inflammasome. Accumulating evidence has suggested that inflammasome activation plays an important role in pyroptosis, which involves NLR family pyrin domain containing (NLRP) 3, the most extensively studied inflammasome, and additionally NLRP1, absent in melanoma 2, and interferon gamma inducible protein 16 inflammasome. It has been revealed that CTSB can promote NLRP3 inflammasome activation after LMP (Wang et al., 2021). In addition to the caspase-1-associated canonical inflammasome activation pathway, pyroptosis includes another non-canonical pathway, a caspase-11-dependent pathway. It has been also reported that inhibition of CTSB activity or knockdown of its expression disrupts the activation of caspase-11 and inflammatory cytokines (Hu et al., 2022). It can be seen that lysosomal damage is closely related to the occurrence of pyroptosis.

#### 3.2.4 Ferroptosis

Ferroptosis is a novel non-apoptotic cell death resulting from the iron-dependent accumulation of toxic peroxidized lipids. Lysosomes exert an important role in iron homeostasis and mainly contribute to ferroptosis through three pathways: autophagy activation, release of lysosomal enzymes, and accumulation of lysosomal iron or nitric oxide (NO) (Chen X. et al., 2021). In terms of autophagy activation, nuclear receptor coactivator 4-mediated ferritinophagy increases intracellular Fe^2+^ and lipid peroxidation levels, resulting in ferroptosis (Hou et al., 2016). Additionally, ferroptosis can also be triggered by autophagy-mediated lysosomal degradation of other proteins, such as aryl hydrocarbon receptor nuclear translocator like through the pathway termed clockophagy, and glutathione peroxidase 4 (GPX4) *via* CMA (Wu et al., 2019; Yang et al., 2019). Lipid droplets increase the supply of free fatty acids required for lipid peroxidation following ferroptosis and facilitate the degradation of GPX4 during CAM to increase lipid peroxidation, both of which result in ferroptosis (Bai et al., 2019; Wu et al., 2019). Sequestosome 1 can induce ferroptosis by increasing cellular levels of both Fe^2+^ and polyunsaturated fatty acids (Yang et al., 2019; Li et al., 2021). It has been shown that LMPs, especially CTSB, can also promote ferroptosis (Gao et al., 2018). Lysosomal membrane permeabilization may further exacerbate free radical production and affect the synthesis of glutathione (a reducing cofactor of GPX4), which in turn reduces the GPX4 activity, ultimately leading to oxidative damage and rupture of the cell membrane (Rizzollo et al., 2021).

### 3.3 Mechanisms of lysosomes in regulating cell metabolism

The metabolic functions of lysosomes are mainly realized through hydrolytic enzymes such as proteases, lipases, nucleases, etc. (Perera and Zoncu, 2016). These enzymes hydrolyze macromolecules and transport nutrients to the cells through specialized channels in the lysosomal membrane (Sagné et al., 2001) Lysosomes act as the cellular energy sensor and regulation center and its specific localization in cells is also regulated: there are mainly two localization statuses, juxta-nuclear position, and peripheral position. Lysosomal trafficking is accomplished *via* distinct transport complexes in two directions: RAB-7/RAB interacting lysosomal protein is responsible for the trafficking in a juxta-nuclear direction; while ADP ribosylation factor like GTPase 8B/SKIP confers translocation toward the cell peripheral position. Adenosine 5‘-monophosphate-activated protein kinase (AMPK) and mTORC1 are two key kinase complexes that sense cellular nutrient levels, regulate metabolic balance, and modulate cell growth, both of which are opposite and complementary (González et al., 2020). Activation of mTORC1 requires the co-stimulation of nutrients, energy, and growth factors, all of which are indispensable. S-adenosylmethionine sensor upstream of mTORC1 is a sensor for methionine, Sestrin2 for leucine, and cytosolic arginine sensor for MTORC subunit for arginine (Rebsamen et al., 2015; Wang et al., 2015; Lawrence and Zoncu, 2019). When cells sense abundant nutrients in these sensors, growth factors induce the phosphorylation of protein kinase B (AKT) and negatively regulate TSC complex subunit 2, activating the Rheb GTPase (Gwinn et al., 2008; Merchut-Maya and Maya-Mendoza, 2021). Amino acids can activate Rag GTPases by altering their binding to guanine nucleotides (Lin and Hardie, 2018). Upon activation of Rag GTPases, RagA/B binds to GTP, and RagC/D binds to GDP (Cao et al., 2021). Activated Rag GTPases can specifically bind to Raptor, a component in mTORC1, recruit mTORC1 to lysosomes, and activate mTORC1, thereby promoting cell metabolism (Saxton and Sabatini, 2017). Interestingly, mTORC1 stimulates the anabolism of the cells with high nutrition, whereas AMPK induces the catabolism of the cells with low nutrition (Jia and Bonifacino, 2019). Activation of AMPK involves a canonical pathway and a non-canonical pathway. In the canonical pathway, the recruitment of AXIN and liver kinase B1 (LKB1) to lysosomes leads to the dissociation of mTORC1 from the membrane surface, and LKB1 phosphorylates AMPK, thereby inhibiting catabolism and cell growth, which can be facilitated by adenosine monophosphate (Sarbassov et al., 2005; Weber et al., 2020). In the non-canonical pathway, AMPK activation also requires the involvement of the framework protein AXIN. Glucose insufficiency and a decline in the levels of its metabolite fructose 1,6-bisphosphate (FBP) can be sensed by V-ATPase-bound aldolases at lysosomes. Once FBP is insufficient to bind to the aldolase, the aldolase can inhibit transient receptor potential cation channel subfamily V (TRPV) calcium channels on the ER, which converts the low glucose signal into a low calcium signal; meanwhile, TRPV can simultaneously bind to the V-ATPase on adjacent lysosomes, resulting in a conformational change that the translocation of architectural proteins AXIN and LKB1 to the lysosomal surface activates AMPK on the lysosomal membrane (González et al., 2020). Additionally, lysosomes regulate the metabolism of metal ions such as iron, calcium, and zinc to prevent their accumulation in the cytoplasm and protect against cell damage.

### 3.4 Mechanisms of lysosomes in regulating immunity

Based on the catabolic and degradative capacity of lysosomes, they play an important role in pathogen recognition and related signal transmission, antigen processing and presentation to T lymphocytes. Moreover, it can combine with dendritic cells, which are the bridge between innate immunity and adaptive immunity (Steinman, 2007). In innate immunity, after phagocytotic cells engulf bacteria, they form phagosomes, which combine with primary lysosomes to form secondary lysosomes and degrade the bacteria internalized by phagocytosis by hydrolytic enzymes (Novak et al., 2010). Toll-like receptors (TLRs) that recognize pathogen‐derived molecular products not only exist on the surface of immune cells, but also on the lysosomal membrane (Nakagawa et al., 2004). By restricting TLR signaling to endolysosomes, pathogens are broken down and nucleic acids are released as self-DNA and RNA are disrupted in the external environment. TLRs bind to NA ligands to endolysosome for wound processing, and because of the presence of the molecular chaperone Unc-93 homolog B1, pathogens are broken down and nucleic acids are released as self-DNA and RNA are disrupted in the external environment (Kim et al., 2008). It has also been further demonstrated that several TLRs need to undergo protein hydrolysis (Sepulveda et al., 2009). The activation of TLR9 and TLR7 signaling pathways in dendritic nuclear epithelial cells requires AEP (a lysosomal cysteine endopeptidase) hydrolysis (Ewald et al., 2011). And in macrophages, histone proteases are similarly involved in TLRs activation (Ewald et al., 2011; Jakoš et al., 2019). Alternatively, there is a negative regulation of lysosomal proteases in host defense in the innate immune response. Attenuation of the protease would enhance major histocompatibility complex (MHC) -restricted antigen presentation because it would disrupt T lymphocyte epitopes (Watts, 2022). In adaptive immunity, TLRs on the lysosomal membrane recognize pathogens, stimulate proinflammatory signals on the one hand, and hydrolyze pathogens into antigenic peptides on the other hand. It promotes the activation of MHC II molecules to cluster of differentiation (CD) 4^+^ T cells and B lymphocytes (Balka and De Nardo, 2019; Lind et al., 2022). Antigen-loaded MHC II molecules can enter the endosomal/lysosomal pathway from the cytoplasm via phagocytosis or receptor-mediated endocytosis. MHC II can become more stable after the removal of nine amino acid residues from MHC II interencapsulated bodies (MIICs) by lysosomal protein hydrolysis. MHC II extends MIICs with the aid of Ia-associated invariant chain and directs MHC II transport to MIICs cytosolic, thereby preventing premature peptide binding to MHC II molecules (Maric et al., 2001; Münz, 2016; Keller and Lünemann, 2017). With the assistance of human leukocyte antigen -DM molecular chaperones, lysosomes can reduce MIICs and class2-associated invariant chain peptide (CLIP) fragments, and catalyze the dissociation of CLIP from the binding site of MHC II molecules (Dengjel et al., 2005; Mellins and Stern, 2014). In addition, Lysosomes can degrade immune checkpoints such as programmed cell death-ligand 1 (PD-L1) and cytotoxic T-lymphocyte-associated protein 4 (CTLA-4) and CD70 and other immune checkpoints (Wang et al., 2019; Wang et al., 2020).

## 4 Regulatory effects of lysosomes on tumors and mechanisms of curcumin intervention

**FIGURE 3:**
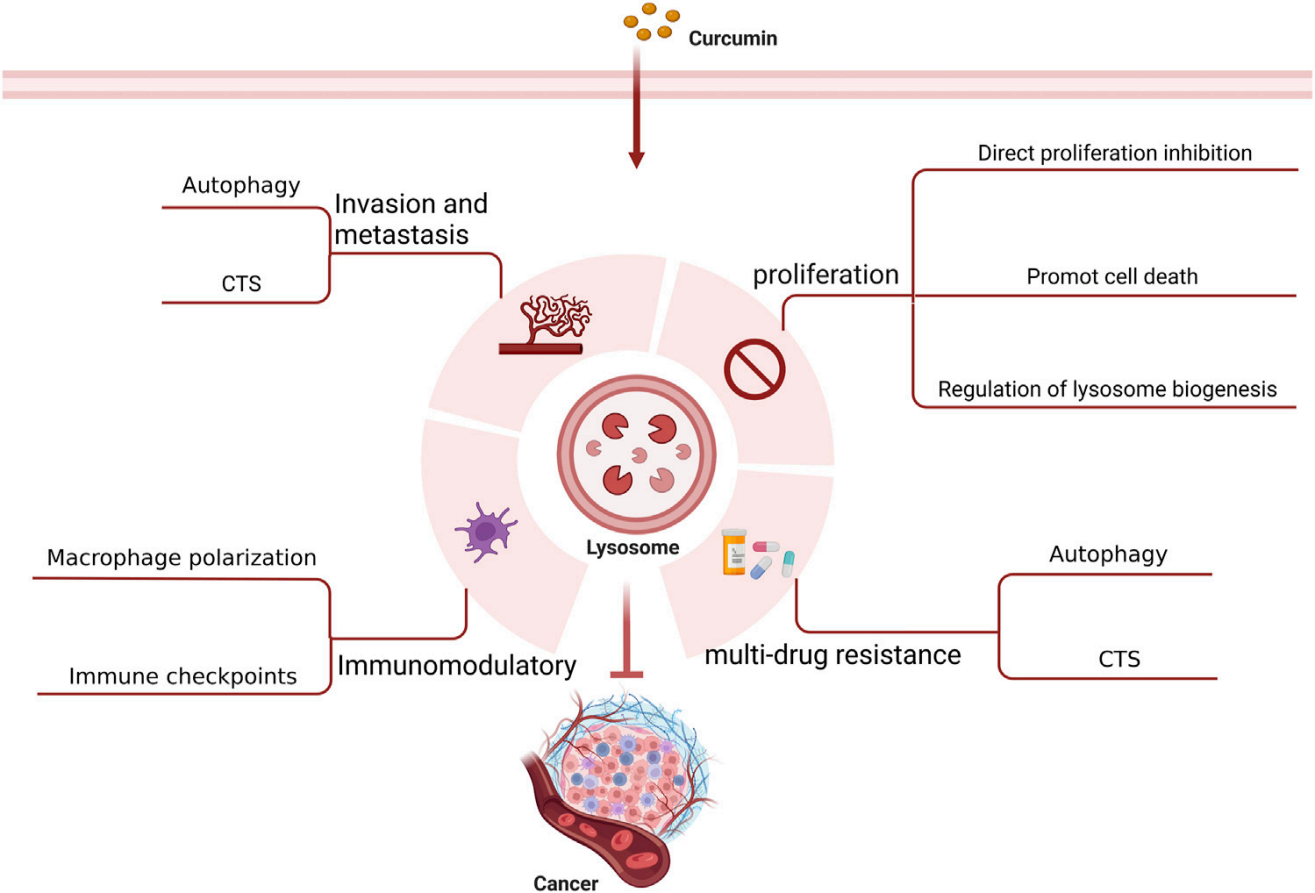
Anti-tumor effect of curcumin targeting lysosomes.

It is believed that lysosomes are critically involved in tumor initiation and progression as well as treatment (Jeger, 2020). Lysosomes can regulate biogenesis, cellular energy metabolism, cell autophagy, programmed cell death, and immunity as well as secrete various hydrolytic enzymes, thereby suppressing tumor proliferation, invasion, and metastasis, reducing tumor drug resistance, and modulating immune function. With an in-depth investigation on the pharmacological effects of curcumin, its anti-tumor effects mediated by lysosome-related pathways have been increasingly validated (Figure 3) (Table 1).

**TABLE 1:** Research progress on the anti-tumor effect of curcumin targeting lysosomes.

Effect		Cancer type	Activities related to lysosome	Ref
Anti-proliferative	Direct proliferation inhibition	-	Affect the assembly of the RNA polymerase I transcription initiation complex at rDNA promoters and inhibits the rDNA promoter activity, resulting in decreased rRNA synthesis	Xu et al. (2020)
	Promote cell death	renal cancer	Downregulate the protein levels of Rictor and Akt while inducing Bax activation and reducing Mcl-1 and Bcl-2 expression	Seo et al. (2018)
		lung cancer, colon cancer	Induced autophagy through lysosomal activation	Zhou et al. (2014), Wang et al. (2016)
		colon cancer, non-small cell lung cancer, breast cancer	Promote tumor cell apoptosis by LMP	Chen et al. (2012b), Khaket et al. (2020), Yuan et al. (2020)
	Regulation of lysosome biogenesis	colorectal cancer, neuroblastoma, Cervical cancer	Regulate the functions of lysosomes to exert anti-tumor activity by directly binding to TFEB and increasing the nuclear translocation and transcriptional activity of TFEB	Zhang et al. (2016a), Song et al. (2016)
Anti-invasive and anti-metastatic	Autophagy	glioblastoma	Induced autophagy	Zhang et al. (2016b)
		non-small cell lung cancer	Suppress autophagic flux in tumors through blocking autophagosome-lysosome fusion	Liu et al. (2021a), Chen et al. (2022)
		oral squamous cell carcinoma, thyroid carcinoma	Activate TFE3-dependent autophagy	Lu et al. (2021), Fan et al. (2022)
	CTS	glioblastoma	Knockdown of CTSL significantly promoted curcumin-induced cytotoxic responses, apoptosis, and cell cycle arrest and also suppressed migration and invasion	Fei et al. (2016)
Reversion of tumor cell multi-drug resistance	Autophagy	non-small cell lung cancer	Suppress autophagic flux in tumors through blocking autophagosome-lysosome fusion	Chen et al. (2022)
		oral squamous cell carcinoma	MTH-3 induce autophagy via TFEB to decrease the viability and trigger intrinsic apoptosis	Tsai et al. (2022)
	CTS	chronic myeloid leukemia	Release CTSB from lysosomes to enhance tumor cell sensitivity to imatinib	Acharya and Sahoo (2016)
Immunomodulatory	Macrophage polarization	glioblastoma, breast cancer	Promote the polarization of TAM to M1 phenotype	Shiri et al. (2015), Liu et al. (2021c)
	Immune checkpoints	head and neck squamous cell carcinoma, liver cancer	Reduce the expression of PD-1/PD-L1 and CTLA-4 immune checkpoint	Zhao et al. (2012), MaruYama et al. (2021)

### 4.1 Anti-proliferative effect on tumor cells

Lysosomes can control tumor proliferation by regulating cell senescence induced by growth factors and relevant oncogenes (Tang et al., 2020). Growth factor signal is initiated at the plasma membrane by receptor tyrosine kinases (RTKs), such as epidermal growth factor (EGF) receptor, which is internalized into cellular structures upon EGF stimulation to promote cell growth. Lysosomes are capable of regulating growth factor signaling through the endocytosis of RTKs (Goh and Sorkin, 2013). Insulin like growth factor 2 receptor (IGF2R) can bind to a regulatory polypeptide IGF2 and be degraded in lysosomes, which inhibits IGF2-mediated tyrosine kinase activation, thus exerting a growth inhibitory effect, whereas M6P/IGF2R is responsible for the trafficking of hydrolases. Studies have documented that M6P/IGF2R is aberrantly expressed in liver cancer (Puxbaum et al., 2012), breast cancer (Iwamoto and Barber, 2007), ovarian cancer (Huang et al., 2006), head and neck squamous cell carcinoma (Jamieson et al., 2003), and prostate cancer (Hu et al., 2006). Aberrant expression of M6P/IGF2R can lead to the mislocalization of hydrolytic enzymes, resulting in the failure to activate the anti-proliferative cytokine transforming growth factor (Gemma et al., 2000). In addition, tumor cells can proliferate indefinitely and evade death. In this regard, oncogene-induced senescence *in vivo* and in cells is an important tumor-suppressive mechanism, where senescence-associated heterochromatin is translocated from the nucleus to the cytoplasm, and tumor cells can degrade the heterochromatin by increasing the number of lysosomes, thus delaying cell senescence (Aird and Zhang, 2015), arresting the cell growth, and inhibiting proliferation.

Infinite tumor cell proliferation requires substantial energy support, while lysosome-mediated regulation can drive tumor cell metabolism. As mentioned above, on the one hand, lysosomes can boost the synthesis of nutrients such as glucose, proteins, and amino acids through the mTORC1 pathway. Loss of GTPase activating protein for RAGA (GATOR1) has been identified in multiple tumors (Bar-Peled et al., 2013). GATOR1 is an upstream key regulatory complex capable of regulating Rag dimer. In breast cancer cells, reduced levels of GATOR1 affect the localization of mTORC1 in lysosomes and attenuate the decrease of mTORC1, thereby facilitating nutrient synthesis and tumor cell proliferation (Chen J. et al., 2018). On the other hand, KRAS-mutant tumors are featured with the ability to grow in a nutrient-deficient and anoxic microenvironment and maintain energy balance (Guo et al., 2011). Increased autophagy widely exists in these tumors and is critical for tumor growth. Lysosomes can degrade intracellular and extracellular substances for cells by autophagy. Macroautophagy can degrade serum albumin and macromolecules into amino acids in pancreatic and lung adenocarcinomas to maintain energy homeostasis in cancer cells (Commisso et al., 2013).

Curcumin can effectively inhibit the proliferation of tumors. Ribosomal DNA (rDNA) can be transcribed to ribosomal RNA (rRNA) to drive cell growth and cell proliferation. mTORC1 is an upstream regulator of rDNA transcription. Curcumin inactivates mTORC1 via repressing mTOR lysosomal localization, which affects the assembly of the RNA polymerase I transcription initiation complex at rDNA promoters and inhibits the rDNA promoter activity, resulting in decreased rRNA synthesis and, ultimately, suppressed protein synthesis as well as tumor cell growth and proliferation (Xu et al., 2020). Due to the excellent anti-tumor ability of curcumin, an increasing number of curcumin derivatives or analogs have been unveiled. As a derivative of curcumin, hydrazinobenzoylcurcumin can promote the formation of autophagolysosomes, induce autophagy, and inhibit tumor cell proliferation in lung cancer A549 cells in a dose- and time-dependent manner (Zhou et al., 2014). The single use of protein phosphatase 242 (PP242), an inhibitor of mTORC1 and mTORC2, cannot induce cell death in human renal cancer cells. However, combined use of PP242 and curcumin can downregulate the protein levels of Rictor and Akt while inducing Bax activation and reducing myeloid cell leukemia-1 and B-cell lymphoma-2 expression, thus accelerating renal cancer cell death (Seo et al., 2018). Oyanguren et al. found that, on the one hand, curcumin induced ER stress and thus caused uncontrollable unfolded protein response and calcium release, which in turn impaired the stability of the mitochondrial compartment and induced apoptosis; additionally, curcumin could mediate the activation of caspase 8 *via* CTS and calpains, which in turn induced LMP, leading to disruption of mitochondrial homeostasis; both two pathways mutually acted to boost tumor cell death (Sala de Oyanguren et al., 2020). Wang et al. (Wang et al., 2016) experimentally demonstrated that curcumin increased the levels of HSP70 and LAMP1 during the autophagy of HCT116 colon cancer cells, which induced autophagy through lysosomal activation, thus leading to cell death. Khaket et al. (Khaket et al., 2020) found that silenced CTSC strengthened the anti-tumor potential of curcumin, and curcumin treatment in colon cancer cells induced ER stress and dysregulation of autophagy accompanied by LMP and ROS generation. This LMP triggered the cytosolic CTSB-mediated BID-dependent mitochondrial membrane permeabilization and thus evoked caspase-dependent apoptosis. Chen et al. (Chen QY. et al., 2012) showed experimentally that curcumin caused LMP and cytosolic relocalization of CTSB and CTSD, which caused lung cancer A549 cell apoptosis. Yuan et al. (Yuan et al., 2020) found that curcumin TPP-PEG-PCL nanomicelles could target the mitochondria of breast cancer cells and escape from the capture of lysosomes, which enhanced its pro-apoptotic effect on tumor cells.

As a master regulator of autophagy-lysosome biogenesis, TFEB has been shown to be activated in a variety of tumors to exert tumor-promoting effects (Calcagnì et al., 2016). Besides, MiT/TFE transcription factors can stimulate the transcriptional activation of the RagD GTPase to regulate mTORC1, thereby accelerating tumor growth (Di Malta et al., 2017). Curcumin can regulate the functions of lysosomes to exert anti-tumor activity by directly binding to TFEB and increasing the nuclear translocation and transcriptional activity of TFEB (Zhang J. et al., 2016). Another study suggested that a synthesized curcumin derivative termed C1 could serve as a novel mTOR-independent activator of TFEB. C1 can specifically bind to TFEB and promote its nuclear translocation, thus increasing the protein levels of LAMP1, LC3B-II, and CTSD in the human neuroblastoma cell line SH-SY5Y; also, C1 can enhance autophagy in N2a mouse neuroblastoma cells and Hela human cervical carcinoma cells through the key autophagy genes, ATG5 and Beclin 1 (Song et al., 2016).

### 4.2 Anti-invasive and anti-metastatic effects on tumor cells

Lysosomes also play a significant role in tumor invasion and metastasis. During tumor invasion, tumor cells can induce the formation of adhesion molecules, and lysosomes can target these specific adhesions, which links the growing tumors to their surrounding environment (Hämälistö and Jäättelä, 2016). The degradation of the extracellular matrix (ECM) is essential for further metastasis. On the one hand, various CTS secreted by lysosomes can degrade ECM, which can not only affect the invasion and metastasis of tumors but also indicate a poor prognosis. Lysosome-secreted CTS can be mainly categorized into cysteine peptidases (CTSB, C, F, et al.), serine peptidases (CTSA and G), and aspartic peptidases (CTSD and E). CTS, which are highly expressed in the majority of tumors, can degrade ECM components such as type IV collagen, laminin, and fibronectin, thereby expediting tumor metastasis (Rudzińska et al., 2019). Interestingly, however, CTSX is highly expressed in the early or pre-malignant stage but lowly expressed in the late stage of several tumors compared with normal tissues, and its underlying mechanism needs to be further investigated (Fisher et al., 2015). On the other hand, ECM components can also be endocytosed by specific cell surface receptors and subsequently degraded through the lysosomal pathway. The lysosomally degraded proteins can supply essential nutrients and energy for tumor invasion. Epithelial-mesenchymal transition (EMT) is a crucial process in tumor invasion and metastasis. Cytokines produced during the EMT process can promote lysosomal degradation of E-cadherin and increase cell motility, consequently promoting tumor cell invasion and metastasis (Wu and Hirsch, 2009). Several suppressors of tumor metastasis such as NM23-H1 can also trigger downregulation mechanisms *via* lysosomal degradation (Fiore et al., 2014). Several regulators have also been shown to affect tumor migration by modulating lysosomal function. EGF mediates anterograde lysosomal movement by activating the p38 mitogen-activated protein kinase pathway, which affects protease secretion and causes tumor cell invasion and infiltration through the basement membrane (Dykes et al., 2017). Activation of TFE3 can promote lysosomal pathway-mediated autophagy in oral squamous cell carcinoma and thyroid carcinoma, which in turn promotes tumor metastasis (Lu et al., 2021; Fan et al., 2022).

In addition, angiogenesis is a key process in tumor growth, invasion, and metastasis. Multiple studies have confirmed that the lysosome-secreted CTSB, D, S, and L also play a vital role in angiogenesis. Upregulation of CTSB expression can increase the activity of vascular endothelial growth factor C and matrix metallopeptidase (MMP) 9, thereby promoting angiogenesis (Yanamandra et al., 2004). CTSD can promote breast cancer angiogenesis by releasing ECM-bound bFGF (Berchem et al., 2002). Active CTSS regulates tumor angiogenesis by controlling the production of type IV collagen-originated anti-angiogenic peptides and laminin 5-derived pro-angiogenic gamma2 fragments (Wang et al., 2006). CTSL acts on the local microvasculature of high-grade serous carcinomas in an autocrine fashion to trigger the transcription and release of carbohydrate-binding protein galectin-1 (Gal 1), hence eliciting tumor angiogenesis (Pranjol et al., 2019). Moreover, RAB7, an LMP, can increase the expression of IL-6 and monocyte chemoattractant protein 1 (MCP-1) through the mTOR pathway, promoting endothelial cell-stimulated melanoma invasion and migration (Zhao et al., 2017).

Curcumin mediates lysosomes to inhibit tumor invasion and migration, which effectively controls tumor progression. Mutated in multiple advanced cancers 1 (MMAC1) is a common tumor-suppressive gene, and curcumin analog C1 can reverse the migration of cholangiocarcinoma owing to MMAC1 inhibition (Jiang et al., 2022). Lysosomes and mitochondria serve as the action sites of curcumin-loaded layered double hydroxide nanoparticles (Cur/LDH NPs); autophagy can enhance the potential of Cur/LDH NPs to inhibit the migration and invasion of A172 glioblastoma cells, the mechanism of which is also confirmed to be correlated with the downregulation of the PI3K/AKT/mTOR signaling pathway (Zhang H. et al., 2016). Curcumin can not only inhibit tumor proliferation by targeting CTS but also affects tumor invasion and migration. Yao et al. (Fei et al., 2016) revealed that knockdown of CTSL significantly promoted curcumin-induced cytotoxic responses, apoptosis, and cell cycle arrest and also suppressed glioblastoma cell migration and invasion, suggesting that CTSL might be a novel target to enhance the anticancer efficacy of curcumin. Another study demonstrated that the derivative of (E)-3-((E)-4-chlorobenzylidene)-5-((5-methoxy-1H-indol-3-yl)methylene)-1-methylpiperidin-4-one (CB-2) was an autophagy inhibitor and blocked the fusion of autophagosomes with lysosomes. CB-2 can increase LC1B-II and SQSTM549 levels associated with autophagosome accumulation in A549 cells, significantly blocking autophagosome and lysosome fusion and thus inhibiting autophagy (Liu Z. et al., 2021). Another derivative of curcumin, (3E,5E)-1-methyl-3-(4-hydroxybenzylidene)-5-(3-indolymethylene)-piperidine-4-one (CUR5g), can also block the recruitment of STX17, a soluble N-ethylmaleimide-sensitive factor attachment protein receptor protein, to autophagosomes through a UVRAG-dependent mechanism, which blocks autophagosome-lysosome fusion; the single use of CUR5g can significantly inhibit the migratory ability and colony formation of A549 cells (Chen et al., 2022).

### 4.3 Reversion of tumor cell multi-drug resistance (MDR)

Chemoresistance is one of the main factors affecting the therapeutic efficacy of tumors, and increasing evidence has shown that lysosomes share a close association with tumor chemoresistance. Hydrophobic weak base chemotherapeutic drugs such as daunorubicin, doxorubicin, lapatinib, vincristine, and nintedanib can be internalized and sequestered by lysosomes through non-enzymatic and non-transporter-mediated mechanisms, thereby preventing them from reaching intracellular target sites and causing tumor MDR (Hurwitz et al., 1997; Groth-Pedersen et al., 2007; Herlevsen et al., 2007; Kazmi et al., 2013; Englinger et al., 2017; Zhitomirsky and Assaraf, 2017). Meanwhile, drugs in the cytoplasm can also be overexpressed inside lysosomes *via* ABC transporters (ATP-binding cassette transporters) such as P-glycoprotein (P-gp) and ABC transporter A3, thereby prompting lysosomal sequestration of chemotherapeutic drugs (Chapuy et al., 2009; Sinha et al., 2018). The differential pH value between lysosomes and cytoplasm is the driving force for this process (Forgac, 2007). Lysosomes cannot sequester drugs forever, and lysosomal exocytosis can also result in tumor drug resistance. Lysosomal exocytosis is a Ca^2+^-dependent process that can be positively regulated by TFEB (Rodríguez et al., 1997; Medina et al., 2011). Lysosomal exocytosis may reduce drug toxicity and availability at the action site, resulting in MDR (Halaby, 2019). Moreover, overexpression of CTS released by lysosomal exocytosis is associated with drug inactivation, and knockdown of CTSL in SKOV3 ovarian cancer cells can increase the pro-apoptotic effect of paclitaxel (Sui et al., 2016).

Importantly, several chemotherapeutic agents or tumors themselves can also affect the biogenesis of lysosomes and alter the number and function of lysosomes, ultimately enhancing tumor drug resistance. Zhitomirsky et al. experimentally demonstrated significant increases in the size and number of cellular lysosomes 72 h after mitoxantrone treatment in MCF-7 breast cancer cells (Zhitomirsky and Assaraf, 2015). For tumors themselves, on one hand, tumors regulate the size, maturation, and activity of lysosomes *via* PI3K (Mousavi et al., 2003). On the other hand, to avoid LMP, tumor cells can overexpress cytosolic protease inhibitors and translocate HSP70 from the cytoplasm to the lysosomal lumen, or promote the binding of HSP70 to an endolysosomal anionic phospholipid bis(monoacylglycero)phosphate and acid sphingomyelinase activity, thereby maintaining lysosomal stability and protecting themselves (Silverman et al., 1998; Nylandsted et al., 2004; Kirkegaard et al., 2010).

The combination of curcumin with anti-tumor drugs can effectively improve the efficacy of anti-tumor drugs and overcome drug resistance. The combination of curcumin derivative CUR5g with cisplatin can significantly enhance the sensitivity of human non-small cell lung cancer cells (A549) to cisplatin by suppressing autophagic flux in tumors through blocking autophagosome-lysosome fusion (Chen et al., 2022). Imatinib mesylate is the first-choice drug for the treatment of chronic myeloid leukemia, yet its clinical application is limited by its therapeutic selectivity and drug resistance. The simultaneous delivery of both imatinib and curcumin *via* nanomaterials induces the release of CTSB from lysosomes, elicits LMP, and promotes cell death, therefore enhancing tumor cell sensitivity to imatinib (Acharya and Sahoo, 2016). Huang et al. constructed a targeting controlled release core-shell nanocarriers based on demethoxycurcumin, which acted on lysosomes and endosomes to form nanoprecipitates and enhanced cytotoxicity, exerting a strong inhibitory effect on anoxic tumor cells with MDR, especially non-small cell lung cancer (Huang et al., 2015). Curcumin can induce excessive mitophagy in thyroid cancer cells, increase lysosomal degradation of mitochondria to trigger autophagic cell death, and promote the redifferentiation of thyroid cancer cells to improve cancer cell uptake of radioiodine-131, finally achieving radiosensitization. Furthermore, ER stress induces high expression of IL-6 and IL-8 to mediate radioiodine resistance in thyroid cancer cells; curcumin as well as the molecularly targeted drug sorafenib can inhibit ER stress to reduce the resistance of thyroid cancer cells to radioiodine (Zhang, 2017). Cai et al. showed that MTH-3, a derivative of curcumin, induced autophagy *via* TFEB to decrease the viability and trigger intrinsic apoptosis in cisplatin-resistant human oral squamous cell carcinoma cells (CAL27) (Tsai et al., 2022).

### 4.4 Immunomodulatory effects

TLRs, an important class of protein molecules involved in nonspecific immunity, mainly exist in the membranes and lysosomal membranes of innate immune cells. Specific TLR3, TLR7, and TLR9 are expressed on lysosomes (Balka and De Nardo, 2019). Autophagy induced by TLR4 or TLR3 activation augments the production of various cytokines such as IL6, CCL2/MCP-1, CCL20/MIP-3α, and VEGFA by promoting TNF receptor-associated factor 6 ubiquitination, which ultimately drives lung cancer progression (Zhan et al., 2014). Currently, researchers have developed a variety of TLRs agonists such as poly (I: C) and lipopolysaccharide, agonists of TLR3 and TLR4, both of which can activate M1 macrophages to produce the anti-tumor effector molecule NO, thus inhibiting tumor growth (Müller et al., 2018). However, CpG ODN, a TLR9 agonist, can enhance the antigen-presenting activity of tumor-associated macrophages (TAMs) and improve the sensitivity to adaptive immune responses (Muraoka et al., 2019). In addition to the impact of TLRs on lysosomes in immune regulation, macrophages exhibit promoting impacts on both phagocytosis and polarization. Upon the uptake of particles by macrophages, massive Ca^2+^ is released from the LMP, transient receptor potential mucolipin 1, which facilitates phagosome formation and increases phagocytic efficiency (Samie et al., 2013). Meanwhile, Ca^2+^ released through the lysosomal Ca^2+^ channel mucolipin-1 (Mcoln1) can activate p38 and NF-κB, realizing M1 polarization of macrophages and ultimately contributing to tumor inhibition (Chen D. et al., 2018). At present, immune checkpoint therapy, as a type of cancer immunotherapy, has attracted increasing attention. It has been revealed that lysosomes can act as major blockers of immune checkpoint molecules because lysosomes can momentarily store immune checkpoint proteins such as CTLA-4, PD-L1, etc (Wang et al., 2020). Among them, CTLA-4 is a lymphocyte surface receptor expressed by activated T cells, and lysosomes can degrade CTLA-4 *via* endocytosis and transfer it to the cell surface *via* the T cell receptor (Chuang et al., 1997; Iida et al., 2000). Tubeimoside-1, a PD-L1 inhibitor, selectively binds to the mTOR kinase target and inhibits the activation of mTORC1, which results in TFEB nuclear translocation and induction of lysosomal biogenesis as well as reduces the abundance of PD-L1 to enhance the cytotoxicity of T cells against tuomr cells, thereby playing an anti-tumor role (Liu X. et al., 2021). Although the correlation of PD-L1 with lysosomes is less well-studied and clearly defined than that of CTLA-4, it is not difficult to discern a close relationship between PD-L1 and lysosomes.

As an immune modulator, curcumin activates the *in vitro* apoptotic activity of tumor cells but has no impact on normal cells within the immune system (Mohammadi et al., 2019). In terms of macrophage polarization regulation, experimental results from Mukherjee et al. showed that either intranasal delivery or intraperitoneal infusion of curcumin relieved glioblastoma and re-polarized TAMs toward a tumoricidal M1-like state (Mukherjee et al., 2016). Shiri et al. found that dendrosomal curcumin significantly reduced the size of metastatic breast cancer and promoted the polarization of TAM to M1 phenotype as well as decreased the levels of M1 phenotype-related signal transducer and activator of transcription (STAT) 4 and IL-12 (Shiri et al., 2015). Moreover, curcumin can regulate tumor immune checkpoints. The results of Liu et al. (Liu L. et al., 2021) demonstrated that curcumin could decrease the expression of immune checkpoint ligands, such as PD-L1, PD-L2, and Galectin-9, in head and neck squamous cell carcinoma and affect tumor invasion by mediating EMT. Also, it was found that curcumin effectively restored the ability of CD8 cytotoxic T cells to lyse tumor cells, boosted T cell proliferation, and increased the production of tumor-infiltrating lymphocytes and effector cytokines, while decreasing the expression of PD-1, TIM-3, inhibitory immune checkpoint receptors, and their ligands in the tumor microenvironment, suggesting re-activation of exhausted CD8^+^ T cell responses. Additionally, this study demonstrated that curcumin reduced CD4^+^ CD25^+^ FoxP3^+^ Treg cells and inhibited the expression of PD-1 and TIM-3. MaruYama et al. also revealed that the curcumin analogue GO-Y030 contributed to inhibiting the anti-PD-1 immune checkpoint and reducing regulatory T cell populations in tumor-infiltrating lymphocytes (MaruYama et al., 2021). Hayakawa et al. unveiled (Hayakawa et al., 2020) that curcumin restored the activity of dendritic cells targeting activated STAT3 and stimulated T cell activity in tumor cells and immune cells to strengthen tumor antigen-specific T cell responses, and synergistic treatment with curcumin and anti-PD-1/PD-L1 improved anti-tumor activity. Similarly, Guo et al. also unraveled (Guo et al., 2021) that the combination of curcumin and anti-PD-1/PD-L1 promoted the immune activity in liver cancer and exhibited superior anti-tumor efficacy. In addition to the PD-1/PD-L1 immune checkpoint, curcumin could downregulate the tumor immunotherapeutic molecule CTLA-4 at both protein and mRNA levels (Zhao et al., 2012).

## 5 Conclusion

Lysosomes are vesicle-like organelles surrounded by a single membrane and containing a variety of acidic hydrolases. The basic function of lysosomes is to complete intracellular digestion through endocytosis, phagocytosis and autophagy, based on which lysosomes are involved in the regulation of cellular autophagy, apoptosis, pyroptosis, ferroptosis and other programmed cell death. Lysosomes participate in the process of digestion is bidirectional. It can not only digest the useless biological macromolecules and aging and damaged cells, but also digest the synthetic nutrients for cells, which is of great significance for maintaining normal cell metabolic activities. Recent studies have shown that lysosomes are also involved in the regulation of innate and adaptive immunity. With the deepening of the research on lysosomes, it is gradually found that the changes and dysfunction of lysosomes are closely related to the occurrence and development of tumors.

Curcumin has received extensive attention as a multi-target anti-tumor drug. The anti-tumor mechanisms of curcumin are diverse, but recent studies have shown that curcumin and its related derivatives can regulate their biological functions by targeting the lysosomal pathway, thereby exerting anti-tumor effects. Curcumin can mediate lysosomes to regulate cellular energy metabolism and lysosome biogenesis, inhibit tumor cell proliferation or promote tumor cell apoptosis, and play an anti-tumor effect. Lysosome itself or various CTS secreted by it can also enhance tumor invasion and metastasis by promoting tumor EMT or tumor angiogenesis, while curcumin can inhibit the above processes. In addition, curcumin can also reverse the MDR mediated by lysosomes and improve the anti-tumor effect of chemotherapy drugs. Finally, curcumin has a positive immunomodulatory effect on the body by regulating lysosome-induced immune dysfunction and immune checkpoint inhibition.

As one of the new targets of curcumin, the mechanism between lysosomes needs to be further explored. Some thoughts on the anti-tumor effect of curcumin on lysosome-related pathways: (1) At present, most studies focus on the anti-tumor effect of curcumin by affecting autophagy, and the research on the regulation of immune pathways is still not systematic and perfect. Moreover, the anti-tumor effect of curcumin directly acts on lysosome-related targets and signal transduction pathways compared with other chemical drugs, whether it has specificity needs to be further clarified. (2) Most of the studies are based on animal experiments or cell experiments, and have not been supported by clinical trial data with a higher level of evidence. (3) At present, the anti-tumor mechanisms of curcumin are mostly based on biological mechanisms. There are few studies on the bioactive metabolites produced after the drug is metabolized *in vivo*, and the biological effects of these substances on the body’s lysosomes still need to be further studied. (4) Lysosomes are heterogeneous, with different sizes and types of hydrolases contained in them, as well as different functions of lysosomes in different cells. (5) In our study, we found that the anti-tumor effect of curcumin through lysosomes could be achieved by either inducing autophagy or inhibiting autophagy. The underlying mechanism of this bidirectional regulation mode needs to be further studied. At present, there is a lack of research on the cellular localization of lysosomes and the mechanism related to cancers. This article summarizes the anti-tumor molecular mechanism of curcumin targeting the lysosomal pathway, and expects more researchers to conduct in-depth research on the targets, gene transcription and modification of curcumin in the lysosomal pathway based on multi-omics technology in the future.

## Figures and Tables

**Table:** 

**AKT**	protein kinase B
**AMPK**	adenosine 5‘-monophosphate-activated protein kinase
**ATG**	autophagy-related
**CD**	cluster of differentiation
**CLIP**	class2-associated invariant chain peptide
**CMA**	chaperone-mediated autophagy
**CTLA-4**	cytotoxic T-lymphocyte-associated protein 4
**CTS**	Cathepsin
**ECM**	extracellular matrix
**EGF**	epidermal growth factor
**EMT**	epithelial-mesenchymal transition
**ER**	endoplasmic reticulum
**ESCRT**	endosomal sorting complex required for transport
**FBP**	fructose 1,6-bisphosphate
**GATOR1**	GTPase activating protein for RAGA
**GPX4**	glutathione peroxidase 4
**GSDMD**	gasdermin D
**IGF2R**	insulin like growth factor 2 receptor
**IL**	interleukin
**LAMP**	lysosome-associated membrane protein
**LEs**	late endosomes
**LKB1**	liver kinase B1
**LMP**	lysosomal membrane protein
**MCP-1**	monocyte chemoattractant protein 1
**MDR**	multi-drug resistance
**MHC**	major histocompatibility complex
**MIICs**	MHC II interencapsulated bodies
**MiT/TFE**	microphthalmia/transcription factor E
**MMAC1**	mutated in multiple advanced cancers 1
**MMP**	matrix metallopeptidase
**NLRP**	NLR family pyrin domain containing
**mTORC**	atypical serine/threonine kinase mechanistic target of rapamycin complex
**PD-L1**	programmed cell death-ligand 1
**PI3K**	phosphatidyl inositol 3-kinase
**PP242**	protein phosphatase 242
**RABs**	targeting GTPases
**RTKs**	receptor tyrosine kinases
**SNAREs**	soluble NSF attachment protein receptors
**STAT**	signal transducer and activator of transcription
**STX17**	syntaxin 17
**TAMs**	tumor-associated macrophages
**TCM**	traditional Chinese medicine
**TFEB**	transcription factor EB
**TLRs**	toll-like receptors
**TRPV**	transient receptor potential cation channel subfamily V
**ULK1**	autophagy activating kinase-1
**V-ATPase**	vacuolar H + -ATPase
